# Is participatory leadership conducive to the construction of virtual teams? Based on multi-agent simulation model

**DOI:** 10.3389/fpsyg.2022.1010007

**Published:** 2022-10-12

**Authors:** Ruonan Liu, Zhenyu Huang

**Affiliations:** College of Public Administration and Humanities, Dalian Maritime University, Dalian, China

**Keywords:** virtual teams, multi-agent model, participatory leadership, cooperation-competition balance, trust

## Abstract

In the Post-COVID-19 Era, with the continuous improvement of the technical level, virtual teams are constantly evolving, and the relationship between leadership and the construction of virtual teams has received more and more attention. It is of great significance to explore the influence of participatory leadership on the construction of virtual teams from a psychological perspective by building a multi-agent simulation model. Based on a simulation platform of NetLogo, the results showed that (1) Participatory leadership is conducive to the expansion of the scale of virtual teams by providing greater space for the development of the members of virtual teams and meeting the team members’ requirements of planning and promotion in the environment, which is decentralized and non-authoritative. (2) However, losing management is not conducive to building a reasonable structure of team members under participatory leadership. (3) The scale of virtual teams and the efficiency of the virtual teams all depend on the relationship between participatory leadership, organizational trust, incentive mode, and the balance between cooperation and competition.

## Introduction

In the Post-COVID-19 Era, the way people travel, work, and study have all changed dramatically, and the Internet space has gradually become the main field and the main position of human activities (Lv, 2021). At present, many enterprises also adopt the method of “virtual team” to divide work, and the relevant research on the construction of virtual teams has become a hot topic in current research, although the current literature on the transition from traditional teams to virtual teams has not been well-outlined and disseminated. However, efforts to understand virtual teams and their effectiveness are growing at an increasing pace (Stratone et al., 2022).

American scholars Miles and Snow (1986) first proposed the concept of virtual teams, believing that virtual teams are “the evolutionary form of networking.” Other scholars constantly complement the concept. For instance, Griffith and Neale (2003) believe that a virtual team is a typical task team that relies on the support of modern network communication media to overcome obstacles across time, space, or organizational boundaries, and ultimately achieve a common mission. Zheng (2020) believes that virtual teams refer to the interaction generated to achieve common organizational goals with members that have complementary skills in different fields on the basis of the development of modern communication technology and interactions. Kankanhalli et al. (2006). believes that increasing globalization and advances in communication technology have fueled the emergence of global virtual teams. In general, the existing literature has the following two consensuses on the concept of virtual teams: on one hand, the background of virtual teams is technological development and progress, while on the other hand, virtual teams are characterized by virtualization, cross-regional collaborative operation, clear goals, and flexible and rapid response.

At present, the research on virtual teams mainly focuses on their innovative performance issues, the staff composition of virtual teams, the management style of virtual teams, the motivation of virtual team members, how to enhance the sense of cooperation of virtual team members, and so on. Limitations in the existing studies can be found by combing the existing literature, as follows:

(1) The evaluation Angle of the development level of the virtual team is too single and limited to the research of the team performance level: Yu et al. (2022) study the impact of team fairness atmosphere on the virtual team performance. Vãtãmãnescu et al. (2022) expanded the relationship between effective communication, team culture strength, and the team performance level in virtual teams, while adding new knowledge about the relationship between team performance level and teamwork satisfaction. Xue et al. (2022) study the impact of knowledge sharing on virtual team performance. Based on the research on the construction of virtual teams, this paper evaluates the team development level from the perspective of team scale and team efficiency.
(2) Participatory leadership, as a democratic leadership style, is more conducive to improving team cohesion than the traditional leadership style (Lin et al., 2019), that is, to maintain the stability of the team. Almaslukh et al. (2022) take the example of the Saudi Arabia banking industry to find the significant positive impact of participatory leadership, training and development, information and communication, and selection and appointment on employee job satisfaction. Ali et al. (2022) propose that participatory leadership influences the generation and orientation of team service goals, ultimately leading to the improvement of team performance. In addition, the influence of participatory leaders on the generation and orientation of team service goals is positively regulated by the inclusive atmosphere of the team. However, there are only a few related studies on virtual teams and participatory leadership; moreover, some research problems have not been further explained in the existing literature. For instance, does a virtual identity exacerbate the crisis of trust that the virtual organization itself has a crisis of confidence? Will decentralization and deauthority make virtual organizations without superior and subordinate relationships more diffuse? Is participatory leadership suitable for virtual teams? How to build an efficient virtual team with mutual trust? Will material or intrinsic incentives become more effective for virtual members? Based on the existing research, this paper discusses the influence of the psychological state of the virtual members on the virtual team under participatory leadership, and emphasizes the importance of organizational trust, participatory leadership, the incentive mode, and the balance between cooperation and competition in the construction of the virtual teams.
(3) Almost all relevant studies are cross-sectional and case-combination research methods, lacking in the development of the virtual team from the time dimension. Although the research content for the virtual teams is very rich, a few works study the development of the virtual teams in the way of multi-agent modeling from a psychological perspective, and our research is to supplement and develop the previous research content.

To make an analysis of the above problems, the remainder of this paper is organized as follows. Section “Literature reviews” reviews the literature related to participatory leadership and the construction of virtual teams. Section “Methods and models” presents the building of a multi-agent model used for NetLogo simulation analysis. Section “NetLogo simulation and analytic results” presents the simulation analysis results of the multi-agent model. Section “Conclusion” presents the conclusion, some policy suggestions, research significance, research limitations, and future research directions.

## Literature reviews

### Participatory leadership and the construction of virtual teams

We believe that it is of unique significance to explore the construction of virtual teams from a psychological perspective. The integration of virtual team members needs to be considered from a psychological perspective, as individual psychological needs are more likely to be amplified in a virtual environment. The role of participatory leadership in the construction of virtual teams is analyzed from a psychological perspective. First of all, groups can be divided into two forms: social groups and psychological groups. Social groups refer to concrete groups with which people can interact face-to-face, while psychological groups refer to a group in a categorical form (Chen, 2006). Virtual teams are the organizational forms of integrating social groups and psychological groups, but more inclined to the form of psychological groups, as it is a group with virtual members that even if it realizes face-to-face communication under virtual space, “real” builds on the basis of “virtual.” Therefore, the virtual teams under the participatory leadership focus on the construction of psychological groups and the members of the satisfaction with psychological needs.

Social cognition theory provides a theoretical tool for us to analyze the relationship between leadership style and the construction of teams from a psychological perspective (Li Y., 2021). Social cognition theory holds that the ternary interaction relationship between mutual influence and interaction is formed among individual cognition, individual behavior, and the external environment. Li Y. (2021) found that a negative leadership style in a working environment affects the mental state of team members (such as the negative psychological state of the employees), and it affects the operation of the entire organization through the behavior of individual employees.

This illustrates the quantity and quality of cognitive effort paid by team members and depends on whether their psychological needs under the guidance of leaders are met in the external leadership environment or not.

The satisfaction of psychological demands further promotes self-control, self-efficacy, and self-worth, thus controlling the degree of self-behavior efforts and promoting the development of the teams. That is, on one hand, the leadership style will affect the change of the mental state, while on the other hand, depending on whether the psychological state is satisfied, will affect the construction of the teams (Yang et al., 2020). Zhou (2019) considers the leadership paradigm that respects the psychological needs of employees’ thinking and that expression can realize the precision and innovation of team decision-making and improve the flexibility and sustainability of the organization in an environment where the life cycle of organizational form and the business model is greatly shortened.

Social cohesion theory argued that the collection of individuals to form a team depends to some extent on whether their mutual needs are met after the union (Li X., 2021). When individual needs are met, individual interactions allow the team to form and sustain. Festinger noted that team members must meet five psychological needs: (1) Attribution needs: the need to live with others. (2) Self-identification and self-esteem needs: who we are, and our personal values and position are determined by our status in various teams. (3) The need to confirm and establish a social reality: the team builds ideas about how things exist and how they work. (4) The need to feel safe and mutually supportive to control anxiety and reduce uncertainty. (5) For its members, the team can meet their needs to solve problems (Li Y., 2021). Therefore, for an individual, the mental state determines his willingness to join the team and work for it. For a team, the psychological state of the team members can facilitate the maintenance of team stability and hence the team performance.

We believe that participatory leadership facilitates the construction of virtual teams if it can meet the conditions, such as the psychological state of daring to express their opinions. The positive psychological meaning is given to the development of the team members and a harmonious and highly cohesive sense of organizational atmosphere. Participatory leadership forms a diversified and inclusive environment, and the leaders share the power. It achieves two-way interaction within the organization. Io other words, leaders not only achieve employees’ voice advice and self-value creation, but also realize the benign guiding role as leaders, and thus they can meet the needs of development value or promotion needs of team members (Zhang M. et al., 2020).

To sum up, thinking about the effectiveness of leadership style from the perspective of psychology, we can see that the participatory leadership style can provide greater development space for virtual team members in a decentralized environment. The upper and lower mobility is strong, which meets the planning needs of team members, and is conducive to the construction of virtual teams. Therefore, we propose hypothesis 1 as follows:

H1: The level of mental state satisfaction of virtual team members mediates the positive relationship between participatory leadership and the construction of virtual teams (team scale and team efficiency).

### Trust in participatory leadership and virtual teams

First, in the perspective of management, trust is an important ability and team intangible asset. Benoit (2003) illustrates that effective team performance was found to be independent of the formation of trust, but trust as a psychological state is a necessary condition for forming a harmonious interpersonal relationship. Information symmetry and good communication distinguish high-performance teams from low-performance teams. If there is no trust, an interdependent interpersonal relationship cannot be established. Once trust is formed, it can reduce interpersonal friction to improve the efficiency of the organization’s operation, maintain the cohesion of the organization, and promote the construction of virtual psychological teams (Yan and Huang, 2020).

Rousseau defined trust as a state of mind willing to take a risk based on positive expectations of the intentions or actions of others (Zhang S. et al., 2021). The sense of being trusted refers to the perception of the trusted on whether he is trusted by others. This perception is a psychological authorization of the active orientation of work roles, and it makes the trusted person feel obliged, capable, and confident to meet the expectations of the person who gives trust to the trusted person. Studies have shown that being trusted triggers the Pygmalion effect, enhances our sense of respect and security, shortens the psychological distance between organizations, and encourages us to show a positive cooperation willingness and cooperation behavior. According to the social identity theory (Zhang M. et al., 2020), under the influence of group identity, people will regulate their own behavior to keep consistent with the group, and one of the core factors that can enhance or weaken people’s tendency to regulate their behavior is the team atmosphere. Zhang M. et al. (2020) also found that an atmosphere full of trust in the team can positively affect knowledge sharing by enhancing the normative behavior of employees, which is the performance of positive cooperative behavior. Therefore, the building of trust will effectively promote collaboration between the virtual team members.

It can be seen from the above findings that the level of organizational trust affects the construction of a team atmosphere and then determines the strength of employees’ willingness to cooperate. Organizational trust can also explain the reflection of interpersonal relationships within the organization. Trust can reduce destructive conflicts, make communication between superiors and subordinates smoother, work more efficiently, and better achieve organizational goals. Team trust promotes coordination and collaboration between teams, and therefore is positively related to team effectiveness (Breuer et al., 2016). Those organizations that lack trust will not only limit the ability of core talents, causing problems such as organizational internal friction, interpersonal tension, and damaged organizational image, but also seriously affect the overall efficient operation of the organization.

When the virtual teams are first built, members usually lack understanding, resulting in a low level of trust in this low-yield and high-risk situation; with increasing knowledge of virtual team members, team members have a deeper understanding of each other’s abilities, knowledge, and attitudes with the help of communication technology. Therefore, trust in a short period of time can be further translated into knowledge-based trust (Lewicki and Bunker, 1996). The rapid trust based on expertise and ability can promote the knowledge-sharing behavior of enterprise employees in a virtual environment and promote the support and help of individuals in the team with each other (Yan and Meng, 2019).

It is worth thinking about the following points: How do you build trust in virtual teams? In a virtual organization, does the trust between team members come from reason, knowledge, or identity? We believe that the trust between virtual team members is still in the first stage or even in a long period of trust in rational judgment, and members focus on a rational trade-off between risks and benefits. Economists believe that trust is based on rational calculation for long-term interests and are unwilling to spend a great number of resources to prevent the result of opportunistic behavior (Zhang H. et al., 2021). Therefore, trust can be quantified, which is the basis of cooperation. Greater trust means that members show more cooperative behavior, and participatory leadership will help to build trust in virtual teams.

Different ways of leadership will affect the construction of the team by affecting the trust relationship between leaders and subordinates. For example, a harmonious and close leadership relationship is an easy way to shorten the psychological distance, strengthen trust, eliminate estrangement, dilute hierarchy, and form a harmonious and unified organizational atmosphere; however, contradiction and conflict in leadership relationships will increase mutual suspicion and doubt that cause organizational division and psychological estrangement making organizational goals impossible to achieve. Leaders and managers who want to be more successful in generating trust among individuals within an organization and helping them to maintain and strengthen their competitive advantage are required to show humility in communication and compassion in behavior (Soderberg and Romney, 2022). Participatory leadership meets the requirements of leaders to create trust among followers. Participatory leadership externally shows a kind of psychological authorization. When employees are satisfied with being trusted and respected, they will strive to act in a way consistent with the expected leadership and improve work performance. Therefore, the sense of respect not only meets the psychological needs of the team members, but also enhances team cohesion, reduces the employee turnover rate, and promotes the development and growth of the teams. Therefore, we propose hypothesis 2 as follows:

H2: The organizational trust of the virtual team members mediates the positive relationship between participatory leadership and the construction of virtual teams (team scale and team efficiency).

### Participatory leadership and motivation methods

All people have desires and demands, and people’s needs are not the same under different conditions. Papac et al. (2020) explain that employee motivation is not only an area of psychological and sociological issues of work and work behavior, but that behavior is also directed toward a goal that instigates needs in a person and the goal is to meet these needs. Only by meeting people’s needs can they play an incentive role in people’s behavior. In today’s highly developed material and spiritual civilization, people’s needs for physiology and safety are increasingly weakened. Their goals are to achieve self-transcendence in order to integrate themselves into the whole society, thus being recognized and respected by society. Therefore, employees have more spiritual needs to pursue spiritual satisfaction based on the virtual world created by spiritual needs. Participatory leadership represents the psychological authorization and the establishment of trust, and trust itself is also a kind of incentive that will encourage the team members to confidently overcome the obstacles in the work and meet people’s emotional communication and “non-material” incentive, which is more conducive to the construction of virtual teams.

However, attaching more importance to spiritual motivation does not mean that material motivation is not important. Team cooperative behavior depends on the desired utility, and cooperative decision is the decision of team members to maximize their expectations after the game with all aspects of risk. When the incentive bonus of team members reaches a certain value, it may lead to vicious competition among members (Cao et al., 2021). Generally speaking, it is generally assumed that, when other people’s work is not good or unsatisfactory, your own days will be easier, and when others’ work is productive, you will be very stressed, and it is not very easy to succeed (Zhu, 2017). It also means that team members begin to form vicious competition in order to obtain the cost that must be owned during the competition. In this case, the key to the formation of organizational cohesion is to effectively resolve conflicts and vicious competition within the team (Lin et al., 2019). Therefore, the material incentives after reaching a certain limit may lead to vicious competition between employees. A certain degree of competition is conducive to the development of the team, but unlimited incentives and excessive competition may evolve into conflicts, which is not conducive to the construction of enterprise team cohesion and the long-term development of the team. The competitive atmosphere within the team will weaken the employees’ willingness to share knowledge and cooperate to some extent, while the interpersonal trust relationship within the team will bring about frequent communication among the members within the organization, thus promoting the employees’ willingness to share knowledge and reducing the cost in the process of knowledge sharing (Zhang J. et al., 2020). Virtual team members have a strong purpose, whether from psychological expectations or material expectations; the cooperation among their members is both affected by trust (psychological expectation) and is also inseparable from the material expectations given by the teams. Therefore, we propose hypothesis 3 as follows:

H3: Limited incentives (material incentives and “non-material” incentives) mediate the positive relationship between participatory leadership and the construction of virtual teams (team scale and team efficiency).

### Balance between participatory leadership and cooperation and competition

Cooperation and competition are two strategies, which are the choice of the behavior subject that decides the relationship between the agent and other subjects in economic activities. The specific strategy depends on the subject’s judgment of the income expectations (Wei and Song, 2013). Yang et al. (2022) found that remanufacturers may choose either cooperation or competition when the patent license fee is exogenous, but will only choose cooperation when the patent license fee is endogenous. With the rapid development of science and technology, the work of human beings is becoming increasingly complex, and most of the work is carried out in a cooperative and competitive environment (Shen, 2020). Cooperation and competition are equally important, because “cooperation” and “competition” are not completely opposite behaviors.

With the advent of the knowledge economy, knowledge has become the most important factor of production. Its fast development speed, superior difficulty, and complex intersection force people to make breakthroughs in the form of cooperation in the process of scientific research. Cooperation and competition theory hold that there are three types of relationships between leaders and employees’ work objectives: cooperative, competitive, and independent. Cooperative work goals enable others to achieve their goals in the process of joint efforts. Cooperative goals lead to collaborative dependencies: people encourage and support each other, share information and resources, constant feedback on their views, strive to help them achieve their goals, and improve their ability to work (Li, 2022). Knowledge sharing based on knowledge cooperation is the most important means for the development of modern science and technology. Constructive conflict in cooperation can enhance the knowledge structure and collective wisdom of researchers themselves so as to relieve the pressure of competition. In other words, members perform better, have better relationships with colleagues, and have deeper feelings for the team in a cooperative environment.

Competitive goals contradict the interests of leaders and employees leading to conflicts between them. The competitive relationship caused by competitive goals will bring negative effects. People only focus on the methods that work for themselves and not for each other (Tang et al., 2021). However, conflicts in a team are not necessarily bad things. Competition can provide more motivation for work and enable members to focus on improving their competitiveness for a period of time. Indeed, task conflicts can stimulate discussions among members, facilitate critical assessments of issues and alternatives, and may lead to better decision-making, thus teams with highly efficient individuals often aim at the appropriate level of conflict. Therefore, it can be seen that an efficient team requires both collaborators and competitors. Differences and inconsistencies in the team are inevitable, and they can lead to team conflicts (Zhou and Zhao, 2017). Kankanhalli et al. (2006) believe that virtual teams in the context of globalization may lead to both task and relationship conflict because of cultural diversity. The relationship between task conflict and team performance is likely to be contingent upon task complexity and conflict resolution approach. Therefore, if the team leader can flexibly and effectively respond to the deterioration of conflicts, as well as resolve conflicts reasonably, the harm to the development of the teams and the enterprises will be reduced.

To sum up, either an excessive sense of competition or an excessive sense of cooperation may lead to the failure of action. Only when the two attitudes of cooperation and competition are unified to reach a balanced degree can the individual power be maximized and finally achieve self-realization. Participatory leadership gives team members more free space, team members work together with their own different skills and experiences producing complementary advantages, and competition between team members within a certain limit will break the original “mindset,” create new ideas, and constantly keep the creativity and innovative spirit of the team (Ouyang and Liu, 2018). At the same time, the members with a strong sense of belonging and a high sense of responsibility for the organization with high cohesion have a harmonious relationship so that they will have a strong motivation for cooperation. However, due to the low sense of trust under the virtual identity, the team members cannot trust them when they bear pressure and confusion. The communication between each other becomes inefficient, which is not conducive to cooperation between groups. Therefore, we propose hypothesis 4 as follows:

H4: The team scale development and team efficiency level of the virtual team depends on the relationship between participatory leadership, organizational trust, motivation mode, and the balance between cooperation and competition.

### The process of the construction of virtual teams under participatory leadership

**FIGURE 1:**
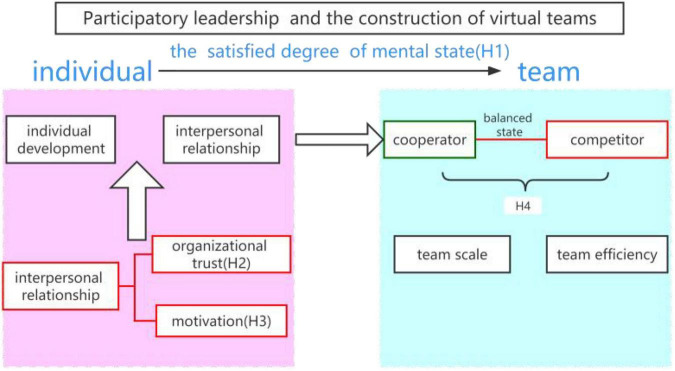
The process of the construction of virtual teams.

First, the leading mode of the virtual teams plays a key role in determining whether the psychological state of the virtual team members is satisfied or not. Participatory leadership in a decentralized and de-authoritative environment provides the development space for the organization members and the mobility of the upper and lower levels is strong, which meets the needs of the team members for their own planning and development, and is conducive to the construction of the virtual teams (H1). Second, the change in the psychological state of the team members cannot be separated from the change in the team’s interpersonal relationship. Organizational trust can explain the reflection of the interpersonal relationship within the organization. The level of trust affects the construction of the team atmosphere, and then determines the strength of the employees’ willingness to cooperate (H2). However, in virtual organizations under virtual identity, an interpersonal relationship may be tenser, which is not conducive for employees to show more willingness to cooperate (H2). Finally, for the problem of the incentive mode, it can be found that the material incentive within a certain limit is conducive to the construction of virtual teams.

(H3) To sum up, the development of team scale and team efficiency level of the virtual teams depend on the relationship between participatory leaders, organizational trust, the incentive mode, and the balance between cooperation and competition. (H4) When the relationship between these four factors can better meet the psychological needs of virtual team members, such as personal development needs or makes the interpersonal relationship of members more harmonious, then team members are more willing to join or stay in the virtual teams. On the contrary, they exit. We present the process of the construction of virtual teams in Figure 1.

## Methods and models

### NetLogo simulation and multi-agent model

NetLogo is now more frequently cited and is becoming increasingly established among researchers in the field. This observation indicates that the field might be evolving into a discipline with shared tools and standards. Along this line, the co-citation analysis depicts two long-term research topics in the field: Opinion Dynamics and Evolution (Hauke et al., 2017). Our research on the evolution of virtual teams also contributes to the enrichment and development of related fields. In fact, scholars have carried out a lot of model studies on the evolution problem of cooperation. For instance, Lavallée et al. (2021) developed a simple individual-based model to test if colony fission and resource allocation may be carried out by workers acting solitarily with no coordination. Jae-Woo (2010) used the Prisoner’s dilemma game model to highlight the roles of cognitively simple agents in the evolution of cooperation who read tags to interact either discriminately or selectively with tolerably similar partners. In addition, they discuss the issue of tag mutability in search of alternative societies in which tag-based parochial cooperation is not only efficient but also robust.

However, the vast majority of optimization and traditional game methods consider cooperative evolution from a local perspective, but the real-world environment is very complex. Various complex factors should be considered to explore the relationship between the individual behavior and the environment (mode) and the interaction behavior between individuals. While NetLogo is a programmable modeling environment for simulating natural and social phenomena based on the Java language, it can be implemented to model complex systems. For example, Hong et al. (2020) studied the evolution of the technology standard alliance (TSA) by using complex adaptive system theory. The echo model is constructed to describe the evolution mechanism which demonstrates the dynamic and complex hierarchical structure of the TSA system. Meanwhile, studying the evolution process from a global perspective needs to consider the overall interests and make a general prediction of the overall development in the future. For instance, Rapin et al. (2017) present a new approach to the study of the immune system that combines the techniques of systems biology with the information provided by data-driven prediction methods. Combining genomic information and simulations of the immune system dynamics, in a single tool, provides new perspectives for a better understanding of the immune system. The NetLogo picture is simple and easy to operate, which can model the complex systems evolving with time, and also provides a reliable method to predict the future development trend. Also, NetLogo is simple enough to operate. Models can be constructed for complex systems evolving over time, and they also provide a reliable way to predict development trends in the future.

Each individual in the model did not behave independently, but mainly worked toward influencing each other. Members of the system are able to interact with other members and the surrounding environment and adjust their own structure and behavior, thus causing changes in the system model, and the model experiences a complex and variable developmental evolution process (Shi and Wu, 2022), Multi-agent modeling and simulation based on the principal modeling techniques change modeling as a whole by building an individual behavior based on the agent, making the model to present a development trend from simple to complex, to describe the construction model, the individual and their mutual behavior of each simulation entity in a complex system, to describe the macroscopic behavior of complex systems by obtaining the emergency of the overall role of the subject through microscopic individuals (Wang et al., 2019), and to reveal the law of the development of complex systems in the real world (Guo and Shen, 2019). In the simulation platform based on multi-agent modeling technology, NetLogo is more suitable for modeling complex systems evolving over time (Zhao et al., 2021).

### The introduction of the cooperation model

#### The concrete introduction of the cooperation model

In NetLogo’s Cooperation model, the turtle image is represented by cows and patches of grass. The green patches (grass) stand two kinds of cows, selfish cows and cooperative cows; cooperative cows show cooperative tendency and altruism tendency, and maximize the sum of their own interest and the sum of the results, whereas, selfish cows show competitive tendency and personal orientation, and focus on maximizing their own interest without considering the results of others, thus maximizing the difference between one’s own interest and others (Zhao et al., 2022). The two cows represent the members adopting the cooperative strategy and the competitive strategy, and are referred to as collaborators and competitors, respectively. They belong to the same organization and need to compete for limited resources (grass) to achieve survival and promotion (reproduction) goals. In virtual organizations, the superior and lower mobility is stronger, assuming that cows that get more grass are easier to breed, and in this sense, people who have easier access to resources are easier to advance and more attractive to other members.

This study explores the outcome of the development of a virtual organization for groups adopting these two strategies under different conditions over time through a NetLogo computer simulation. The place where each member stands is called “tiles,” symbolizing the resources of a unit. Each member of each round uses the resources on the tile he stands on. Once a competitor stands on a tile, it uses up all the resources in that unit of the area regardless of whether the resources are above the growth threshold. Cooperators also use resources, but stop after using a certain level of resources because the supply of resources significantly slows once the supply is below a specific resource regeneration threshold. In brief, cooperators will share personal benefits, trust their teammates, be willing to share resources, and leave more resources (food) for the group. On the contrary, competitors will use full resources regardless of the survival of the whole group, expressing distrust of other members, and it shows that the organizational cohesion is weak. Therefore, the operation of cooperation in this model is defined as restraining the use of resources and stopping at the regeneration threshold. To simplify the problem, it is assumed that cooperators are always cooperative and competitors are always competitive.

#### Reasons for selecting the cooperation model

On the one hand, scholars in related fields have used cooperative models in NetLogo. For example, Geng and Li (2011) designed and implemented a cooperative behavior system based on NetLogo platform, and the effect of task difficulty on cooperative behavior is investigated in this system. Chen et al. (2014) used evolutionary game theory and a multi-agent modeling method, and the asymmetric evolution game and the simulation model of industry-university-research cooperation were constructed relying on the NetLogo simulation platform. The operation of the cooperative system with different revenue parameters was analyzed and verified. Qin et al. (2020) modified the cooperative model to further construct a complex behavioral model for composite entrepreneurial teams of college students.

On the other hand, the Cooperation model can dynamically observe the changes in the team scale at each stage. Therefore, by observing the changes in the number of cooperators and competitors, we can see the influence of the way of team leaders, the effectiveness of the team incentive system, the development and change of team members’ trust in the organization, the degree of team information sharing, and the future development trend of the team, which can accurately grasp the cooperative evolution simulation process of virtual team members. More importantly, the model can simulate the environment of different teams by controlling the variables. Each variable can be given a specific research meaning, so that the Cooperation model can fit better with the relevant theories of participatory leadership and the construction of virtual teams.

### Design of cooperation model

#### Dependent variables

The initial data are set to 20, that is, the total number of members in a team is 20. The research of Lawrence Putnam showed that if a team has more than 20 participants (Boren, 2016), then more effort would be required when compared to a study including only five or fewer participants. Compared to small teams, large teams can take more than five times the time to complete the tasks. Therefore, the team efficiency of small teams is controllable for studying the impact of participatory leadership on the construction of teams, and it is of more significance to study how to build efficient teams of large teams by participatory leadership.

#### Independent variables

(1) Stride-length (team members’ free space): This variable determines the degree of goal realization. By building a common vision and goals, leaders provide the space for everyone to develop freely and push each round to move a distance. When the total value rises, the space for everyone to develop is expanded. Furthermore, it can be considered that the larger the scale of the organization, the more capital and other resources are invested, the more space for development, the default value of 0.08, and the range is from 0 to 0.30. This value is determined by the leadership style, and different leadership methods will set up different degrees of free space. For team members, under participatory leadership, the difficulty in promotion may decrease mean lower promotion costs. Therefore, when the virtual members’ material income may be greater than the expected threshold or even the promotion threshold after subtracting their own consumption cost, it indirectly means that virtual members are highly likely to be willing to join or stay in the virtual teams.
(2) The grass-energy (behavioral gain): Behavioral gain refers to the benefit of achieving the goal. In the virtual world, team members will pay more attention to timely material incentives, whether they bring benefits to the team or not. When the collaborator achieves his work goals, it means that he is familiar with the work environment, has a harmonious interpersonal relationship, and increases the psychological security of the team. Also, with the addition of timely money incentives, they will become more dependent on the organization. But competitors need timely money incentives. By setting that a person adds each additional unit of resources, his competitiveness will increase by one unit, that is, to achieve the common goal of one unit, to master the resources of one unit. The value ranges from 0 to 200 with an initial default value of 51.
(3) Metabolism (consumption cost): Consumption cost refers to the input of behavioral cost, where each person gets resources and pays a certain cost. When it is reduced to 0, the competitiveness means 0 which means leaving the team, the cost metabolic range is 0–99, and the initial default value is six. For the team members, when their own material gain minus their own consumption cost is more than expected to obtain, it shows that the psychological state needs of the team members are met to a certain extent, and they are willing to stay in the virtual teams.
(4) Promotion cost (competitive cost): Each promotion of an individual requires a certain resource cost. This value represents the resource cost required for the promotion. Promotion costs range from 0 to 99, with an initial default value of 54.

#### Controlled variables

(1) Reproduction-threshold (promotion threshold): When a person’s resources or ability reach a certain value, he can obtain the promotion qualification, indicating the scale of the basic amount of resources that an individual must have to obtain the promotion. The promotion threshold ranges from 0 to 200, with an initial default value of 102.
(2) Low-high-threshold (resource regeneration threshold): The value range of the resource regeneration threshold is 0–99, and the initial default value is nine. The resource regeneration threshold is set in the range of 0–99, where the resource regeneration is calculated as “high resource regeneration probability,” and above the resource regeneration threshold, it is calculated as “low resource regeneration probability.” Low resource regeneration probability (low-growth-chance) is the percentage of resources able to be regenerated below the regeneration threshold. The high resource regeneration probability and the low resource regeneration probability range from 0 to 99, with an initial default value of 77 and 30, respectively. The larger the value, the smaller the behavioral difference between collaborators and competitors. High resource regeneration probability refers to the percentage of resources above the regeneration threshold that can be regenerated. The smaller the value, the smaller the behavioral difference between collaborators and competitors.
(3) Max-grass height (maximum resource input): Set the maximum number of resources, with an initial default value of 10.
(4) Cooperative probability (organizational trust): Range: 0–1.0, and the initial default value is 0.5 (see Table 1 The cooperation model-related parameter settings).

**TABLE 1:** The cooperation model-related parameter settings.

Main parameter	Parameter meaning	Parameter values range	The initial setting of parameters
Initial-cows	Team members	0~100	20
Stride-length	Team members’ development space	0~0.30	0.08
Grass-energy	Behavioral gain	0~200	51
Metabolism	Consumption cost	0~99	6
Promotion cost	Competitive cost	0~99	54
Reproduction-threshold	Promotion threshold	0~200	102
Low–high–threshold	Resource regeneration threshold	0~99	9
Low–growth–chance	Expected threshold	0~99	30
High–growth–chance	High-resource-regeneration-probability	0~99	77
Max–grass–height	Maximum-resource-input	0~40	10
Cooperative probability	Organizational trust	0~1.0	0.5

### The running logic of the model of cooperation

**FIGURE 2:**
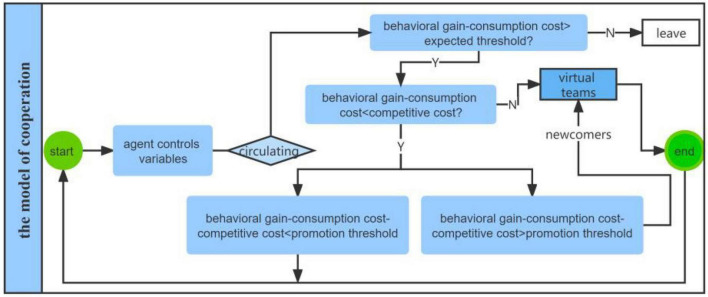
Schematic diagram of the running logic of the model of cooperation.

The Cooperation model mainly presents two key procedures: (1) The system operation constantly changes the composition of team members, and with each operation of the model, the members will consume their own energy (cost) and gain behavioral benefits. If the difference between revenue and cost is greater than the minimum expected threshold of the team, the team members can stay in the team, otherwise they will exit. (2) The team scale will constantly change during the operation of the model. The difference between the income and the cost of the team members is greater than the expected threshold, and the cost consumed by the team members is greater than the competitive cost, which means the entry of new members and the expansion of the team scale (see Figure 2).

## NetLogo simulation and analytic results

**FIGURE 3:**
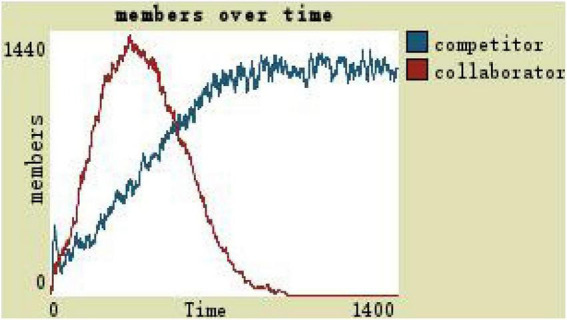
Team members change at the initial values.

With the initial values in the case, as can be seen from Figure 3, collaborators first increase and then reduce, and competitors are in a state of constant growth. Next, by changing the way of leadership, the team organization trust, and leadership incentive way, three dimensions constantly repeated two key procedures at the same time while observing and comparing the number of collaborators and competitors to determine the evolution of the virtual teams (see Figure 3).

### The effect of leadership style on the construction of virtual teams

**FIGURE 4:**
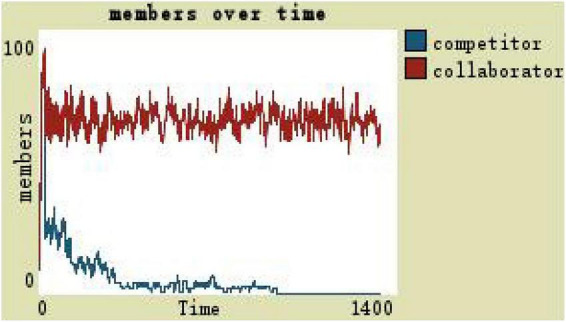
Changes in team members with the participatory leadership.

The scope of the development space of the organization members is taken as the independent variable and the others are taken as the dependent variables. When the development space of the organization members is zero, it indicates that the leadership style of the team at this time is an all-round autocratic leadership without gaps, and the team members’ development space is small. On observing and comparing the virtual teams in the autocratic leadership of a number of collaborators and competitors (see Figure 4), it can be found that in the process of changing the number of collaborators and competitors under the autocratic leadership style, there are always more collaborators than competitors in the middle and late stages, but the overall scale of the team fluctuates around 124.

**FIGURE 5:**
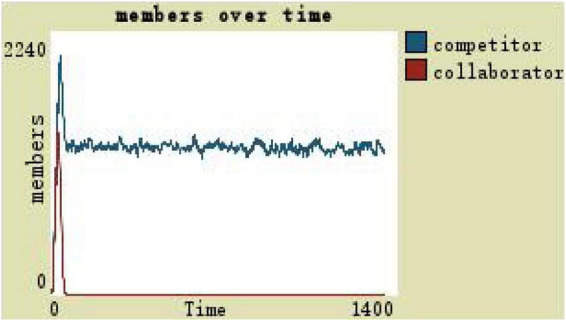
Changes in team members with the participatory leadership.

If the development space of set organization members is 0.3, that is, simulation of participatory leadership, it means that the team members are in the background of a large amount of resources and large development space, running results as shown in Figure 5. At the high level of development space, the number of competitors is more than the collaborators, and the team scale remains at around 1,300 in the later stage (see Figure 5).

### The impact of organizational trust on the construction of virtual teams

**FIGURE 6:**
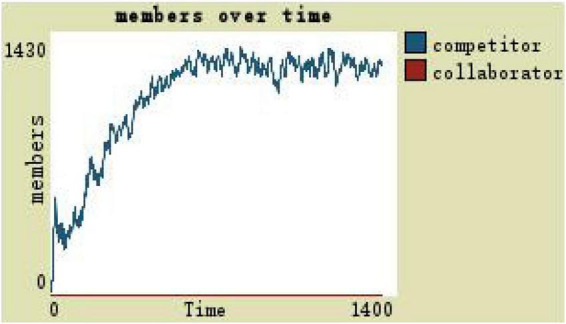
Team members change with the low level of trust.

Observe the number of team members with the organization trust as the independent variable and the other variables remaining unchanged. The trust level of 0 indicates that the virtual team is in a state of extreme distrust, and the trust level determines whether the team members adopt a cooperative or competitive resource-use approach. Changes in team members under the low level of trust are shown in Figure 6: competitors are in a fluctuating growth state, the number of collaborators is always 0, and the overall team scale remains around 1,400 (see Figure 6).

**FIGURE 7:**
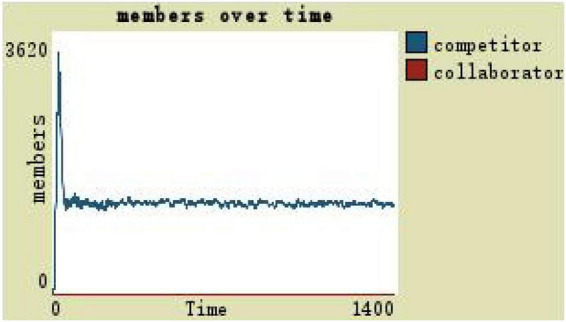
Members change in low-trust team with the participatory leadership style.

If by controlling the scale of the organization of the development space, when the level of trust is 0, development space is 0.3, and observed changes of team members with a low level of trust under the participatory leadership are as shown in Figure 7, competitors reached 3,010 at 49 steps and then began to decline, from 54 to the end at around 1,200, the number of collaborators is always 0, and the overall scale of the team remains at around 1,400.

**FIGURE 8:**
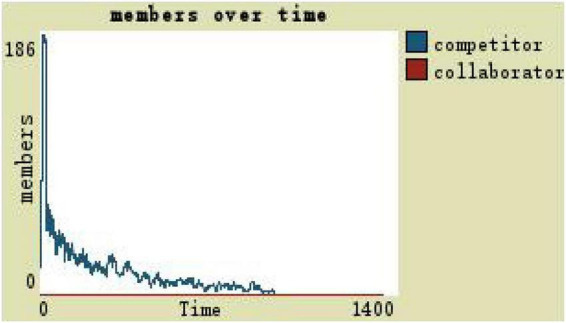
Members change in low-trust team with the participatory leadership.

If the trust degree is 0 and the development space is 0, team members with a low level of trust under autocratic leadership are observed as shown in Figure 8: competitors will initially have a small growth and then gradually decrease, and collaborators will remain at zero. In the medium term, the team scale is around six (see Figure 8).

**FIGURE 9:**
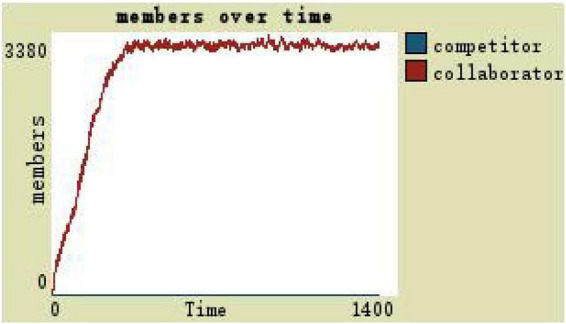
Team members change with high level of trust.

When the trust of the organization is 1 and the team is highly cohesive, the change in the team members is as shown in Figure 9: the number of collaborators continues to grow and stabilize, and the overall scale of the team remains around 3,400 (see Figure 9).

**FIGURE 10:**
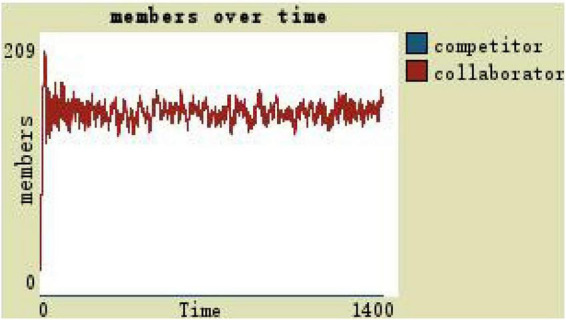
Members change in high-trust team with the participatory leadership.

With the trust degree is 1 (the team is highly cohesive) and the development space is zero, the total team members are up to 200, and the number of collaborators, that is, the team scale, is maintained at around 140 (see Figure 10).

**FIGURE 11:**
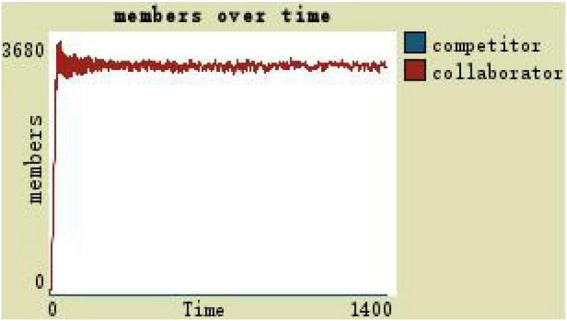
Changes with team members in the high level of trust with the participatory leadership.

How would the scale of the team ever change if one member somehow increased the level of trust among virtual team members in some way under participatory leadership? For example, if the trust degree is 1, and the development space is 3 (see Figure 11). The competitors were always 0, the collaborators reached 3,730 at 52 steps, and then the number fluctuated around 3,200.

### The impact of incentive styles on the construction of virtual teams

**FIGURE 12:**
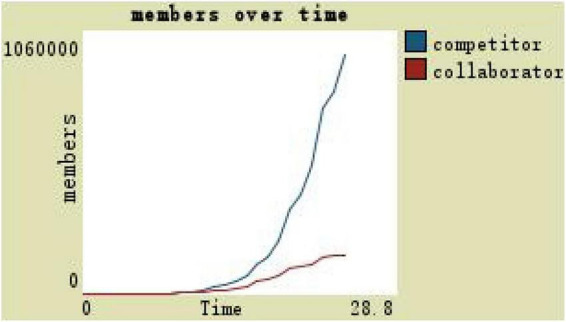
Changes in team members at high incentive level.

When material incentive was used as an independent variable, other variables remain unchanged, the team material incentive reached the maximum, and the important influence on the construction of the team can be observed under the high incentive level of team members change as shown in Figure 12: competitors always more than collaborators, the total number of teams reached more than 400,000 from the beginning, and material incentives affect competitors in particular (see Figure 12).

**FIGURE 13:**
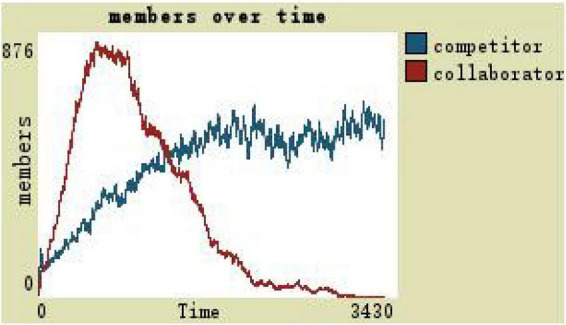
Team members change with the low level of incentive.

When the material incentive is 0 and members’ behavior gain below the initial value (that is, the team member gain is set at 30), team member change under the low level of incentive as shown in Figure 13: Figure 13 depicts the overall trend similar to the initial value of team members changes in Figure 3, but the total team scale maintained at around 1,000, which is far below the team scale under the initial value (see Figure 13).

**FIGURE 14:**
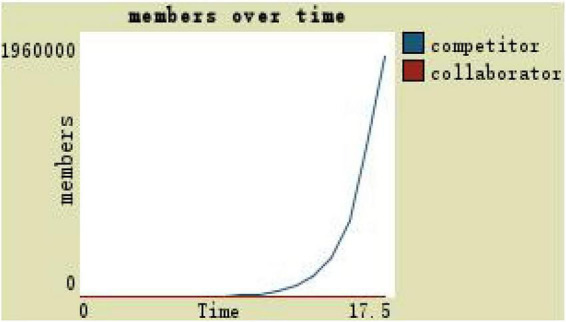
Team members change with participation leadership of highly motivated and low-trust level.

If under the participatory leadership in a decentralized environment, the development space is 3 and the trust degree is 0, then it can be observed with a timely and maximum material incentive in Figure 14: the number of competitors reaches 1.85 million and the collaborator is 0. Even under the maximum material incentive, the collaborator is still at 0 and the team scale reaches the largest, but the composition of team members is obviously unreasonable (see Figure 14).

## Conclusion

### Main findings

(1) By comparing the simulation results shown in Figures 4, 5, 7, 8, 10, it is found that the scale of the virtual teams under participatory leadership is much larger than that under autocratic leadership, which proved that in the case of participatory leadership with large space for development, participatory management, as a democratic management mode, is applied to the construction of virtual organization, which can enhance the sense of responsibility of employees and give employees more free space to develop and fully mobilize the enthusiasm of work. At the same time, the employees can have more opportunities to participate in the management activities forming a structure that is decentralized. This adapts to the construction characteristics of virtual teams. Thus, hypothesis 1 is partly proved in this finding that the degree of mental state satisfaction of virtual team members positively mediates the relationship between participatory leadership and virtual team scale.
  It is also found that, as shown in Figure 11, in the case of participatory leadership and high organizational trust, the team size is the largest, while the composition of team members is unreasonable, which is not conducive to the long-term development of the team. Thus, hypothesis 2 is inversely proved that the organizational trust of virtual team members mediates the negative relationship between participatory leadership and virtual team efficiency. It is possible because being trusted may also be a burden, which means that too much trust may lead to the emotional exhaustion of employees and result in the reduction of their work performance. Thus, it is equally important to consider the dispersion in trust and monitoring that exists within teams (De Jong and Dirks, 2012). When individuals have a high job autonomy along with reduced regulation, too much trust can be detrimental to work performance, because too much trust and too little regulation may worsen job performance due to social inertia (Sun et al., 2018).
(2) By comparing the results shown in Figures 4, 5, it is found that different leadership styles have different impacts on the team composition. In the team composition, the number of competitors has an obvious advantage, indicating that the competition among the virtual teams is very fierce under participatory leadership. There are even cases where the number of collaborators in a virtual team is almost zero, which shows that too loose management has a negative impact on the development of the collaborators. Too much development space, too loose management, and too many competitors are not good for the collaborators entering the team. In order to obtain more promotion resources, competitors should carry out vicious competition and seize the living space of competitors. Competitors will be in a balanced state after fierce competition, but too many competitors in a team are unreasonable for the team composition, which is not conducive to the formation of the optimal team, as well as their later development. Fully democratic leadership does not provide enough social support to the team members and may also increase their work anxiety, which is actually not conducive to the personal development of the competitors (Li et al., 2022). Thus, hypothesis 1 is partly inversely proved that the degree of mental state satisfaction of virtual team members negatively mediates the relationship between participatory leadership and virtual efficiency.
(3) In the virtual space, with the increasing sense of distrust brought by the virtual identity, it is found that, by comparing the results shown in Figures 6, 9, the trust of the organization has little influence on the competitors, but it is not conducive to the survival of the collaborators, indicating that the collaborators pay more attention to the psychological security and trust brought by the team.
(4) By comparing the results shown in Figures 12, 13, it is found that the team scale under the timely and low-delay rewarding with the high incentive level is much larger than the team scale under the low incentive level, which shows that the timely material incentive is beneficial to the construction of the virtual teams. Figure 14 also shows that even if participatory leadership with high incentive levels is conducive to the team scale, it is not conducive to the efficient operation of the team. From (3) and (4), it can be concluded that organizational trust is of obvious significance to the reasonable composition of team members. Thus, hypothesis 3 is proved that limit incentives (material incentives and “non-material” incentives) positively mediate the relationship between participatory leadership and virtual team development (team scale and team efficiency).
(5) By comparing the results shown in Figures 1–14, we found that if we want to maximize the virtual team scale and achieve efficient team operation, teams need both cooperation and competition, and a balance between organizational trust, the development space of the team, and spiritual or material incentives. Thus, hypothesis 4 is proved that the team scale development and team efficiency level of the virtual team depend on the relationship between participatory leadership, organizational trust, motivation mode, and the balance between cooperation and competition.
  In short, to maximize the virtual team scale and achieve efficient team operation, on one hand, participatory leadership is conducive to the expansion of the team scale, but the composition of the team members under participatory leadership is not reasonable. Too much free development space is beneficial for the development of team competitors, and it occupies the living space of collaborators. Participatory leadership needs to set a free team development space to a certain extent. On the other hand, teams need both cooperation and competition, and a balance between organizational trust, the development space of the team, and spiritual or material incentives.

### Management implications

(1) Participatory leadership and the construction of virtual teams. Indeed, participatory leadership of excessive development space may lead to vicious competition, and companies need to set a certain development space, such as setting the red line and the bottom line (Su, 2022). Team members cannot break the bottom line, and virtual teams need rules, strengthen supervision, and encourage innovation in parallel and seize the opportunities brought by the virtual era at the same time. Companies should also timely introduce corresponding regulations to restrain and prevent. The virtual members should be constrained at technical, moral, and ethical levels, and the supervision mode should be clearly defined.
(2) Organizational trust and the construction of virtual teams. The low trust level of virtual teams by virtual identity is not conducive to the development of virtual team collaborators (the reasonable composition of the team), so the construction of virtual teams needs to strengthen the trust between members. For example, organizing virtual space induction training can strengthen face-to-face communication and solve conflicts within the organization to a certain extent through effective communication, especially cognitive conflict which can also enhance the sense of trust among members (Zhang S. et al., 2021). Meanwhile, in leadership activities, leaders should, especially, pay special attention to their subordinates’ trust in themselves, because high trust can make their subordinates produce high organizational identity and organizational loyalty, and stimulate conscious organizational civic behavior. Leaders should take the initiative to approach their subordinates with a sincere heart, think about problems from their psychological needs, and create a good relationship from the overall organizational situation, rather than starting from personal preferences and personal interests and highlighting personal intimacy, to make “small circles” and gangs (Soderberg and Romney, 2022).
(3) “Non-material” incentive and the construction of virtual teams. The impact of material incentives on team competitors is very obvious. However, in today’s highly developed material and spiritual civilization, for the team competitors, simple material incentives are essential. Meanwhile, it should also meet the competitors’ attention toward their self-status and self-value psychological needs. Through promotion, such as position or professional title (Li X., 2021), an effective promotion and promotion mechanism can be constructed to provide a promotion possibility for each team member, further mobilize the enthusiasm of competitors, and also attract new recruits and promote the expansion of the team scale. If only developing the promotion mechanism, to promote the expansion of the team scale may also cause unreasonable team composition; therefore, for the company’s internal collaborators, paying attention to personal development, as well as paying close attention to team relationship, and trust incentive can significantly improve the team performance. Therefore, the team can play the role of cultural incentive and create a harmonious and good team culture atmosphere, so as to promote the realization of the work value of the staff within the team.
(4) Balance between cooperation and competition and the construction of virtual teams. Companies need to cultivate the competitive consciousness and spirit of virtual team members. Companies clearly show that competition is not a vicious competition, but a unified constructive competition with a common goal (Zhao, 2022). It is not only necessary to continuously strengthen the awareness of constructive competition among the members, but also to carry out organizational collective actions, adjust the organizational interpersonal relationship with the help of emotional management, encourage their mutual support, unity, and cooperation, timely completion of work tasks, and achieve common progress (Zhu, 2017).

### Research contributions

(1) By exploring the influence of organizational trust and the incentive mode under participatory leadership on virtual team construction, we enrich the research on participatory leadership and virtual team construction. At present, the relevant research on the virtual team mainly focuses on the team performance level, and the research results on the team size and team efficiency level are relatively limited, and they mainly use actual cases to explain and lack the attention of visual research means. This study uses the simulation of the control variables to verify the relevant conjecture and put forward the relevant practical and targeted specific suggestions, which have a certain innovation.
(2) The logical discussion from the individual level to the team level not only facilitates a deeper understanding of the construction process of virtual members to a virtual team, but is also a beneficial integration of existing research. Most of the existing studies concern the direct application effects of certain atmospheres in virtual teams and related theoretical deduction. However, this study takes personal development and interpersonal relationship as the entry point from the psychological perspective, which beneficially complements the construction process of a virtual team.
(3) The previous studies related to the team building hypothesis have been tested through the simulation here, such as the relationship between team trust and team performance, and the existing participation and team building research pay attention to the analysis of the positive influence on the team building, and the negative effects of participation leadership on team building lack attention. Through simulation, it is found that loose management under participatory leadership is not conducive to building a reasonable team member structure.

### Limitations and future research directions

(1) Moreover, this paper has some research limitations. For example, the simulation model based on the virtual teams built by NetLogo needs to be further improved, such as investigating the scale of specific teams, digital performance management, the introduction of human-machine collaboration (Tsai et al., 2022), and the impact of the introduction of digital people on virtual teams, so as to make it more consistent with the construction of virtual teams.
(2) Some numerical settings of the cooperation model itself lack reasonable explanations. The research does not combine the visual model with specific case studies, and it is also a future research direction to combine the multi-agent modeling method with the specific investigation and research including a large number of practical cases.
(3) This model only discusses the participation of leaders. This study only explores the influence of organizational trust and the incentive mode on the process of virtual team construction, and whether there are some other variables or processes that can mediate the influence are also worthy for the researchers to consider.

## Data availability statement

The original contributions presented in this study are included in the article/supplementary material, further inquiries can be directed to the corresponding author.

## Author contributions

RL was responsible for manuscript writing and simulation. ZH was responsible for the manuscript guidance and format modification work. Both authors contributed to the article and approved the submitted version.
